# The Narrative Medicine Approach in the Treatment of Diabetic Macular Edema: An Italian Experience

**DOI:** 10.3390/ijerph19159367

**Published:** 2022-07-30

**Authors:** Edoardo Midena, Chiara Polo, Luisa Frizziero, Maria Giulia Marini, Rosangela Lattanzio, Maria Vadalà, Elisabetta Pilotto, Monica Varano

**Affiliations:** 1 Department of Ophthalmology, University of Padova, 35128 Padova, Italy; chiara.polo@studenti.unipd.it (C.P.); lfrizziero@gmail.com (L.F.); elisabetta.pilotto@unipd.it (E.P.); 2 IRCCS—Fondazione Bietti, 00198 Rome, Italy; monica.varano@fondazionebietti.it; 3 Health Care and Wellbeing Area, ISTUD Foundation, 20154 Milan, Italy; mmarini@istud.it; 4 Department of Ophthalmology, Scientific Institute Ospedale San Raffaele, University Vita-Salute, 20132 Milan, Italy; lattanzio.rosangela@hsr.it; 5 Department of Experimental Biomedicine and Clinical Neuroscience, University of Palermo, 90127 Palermo, Italy; maria.vadala@unipa.it

**Keywords:** narrative medicine, diabetic macular edema, diabetes, patient’s journey, quality of life, caregiver

## Abstract

The study retraces the healthcare pathway of patients affected by diabetic macular edema (DME) through the direct voice of patients and caregivers by using a “patient journey” and narrative method approach. The mapping of the patient’s journey was developed by a multidisciplinary board of health professionals and involved four Italian retina centers. DME patients on intravitreal injection therapy and caregivers were interviewed according to the narrative medicine approach. Narratives were analyzed through a quali-quantitative tool, as set by the narrative medicine method. The study involved four specialized retina centers in Italy and collected a total of 106 narratives, 82 from DME patients and 24 from caregivers. The narratives reported their difficulty in identifying the correct pathway of care because of a limited awareness of diabetes and its complications. Patients experienced reduced autonomy due to ocular complications. In the treatment of diabetes and its complications, a multidisciplinary approach currently appears to be missing. DME reduces the quality of life of affected patients. The narrative medicine approach offers qualitative and emotional patient-guided information. The patient journey provides all of those involved in the management of DME with flowcharts to refer to, identifying the critical points in the healthcare journey of DME patients to improve the management of the disease.

## 1. Introduction

Diabetes mellitus (DM) is considered a global epidemic of the 21st century with about 422 million people worldwide affected and 1.5 million deaths globally attributed to diabetes each year [1]. In Italy, diabetes affects more than 3,539,000 people, about 5.9% of the general population, leading to 21.187 deaths [2]. Both the number of cases and the prevalence of diabetes have been steadily increasing over the past few decades. Diabetic retinopathy (DR) is the most common and specific complication of DM and represents one of the leading causes of visual impairment and preventable blindness worldwide in the adult working population [3,4]. Epidemiologic data suggest that at least 30% of the population with diabetes is suffering from DR. Diabetic macular edema (DME) is the major cause of vision loss associated with DR. It is related to some characteristic symptoms, including visual blurring and metamorphopsia, which cause relevant limitations of daily life activities, such as driving or reading. There are approximately 93 million people with DR, including 21 million with DME worldwide [5]. The overall prevalence of DME is 6.81% in people with diabetes, accounting for 12% of new cases of blindness annually [5]. According to studies of the natural history of DME, 24% of eyes with DME will lose at least three lines of vision within three years. The gold standard in the current treatment of DME is the intravitreal injection of anti-VEGF or corticosteroid drugs. The three most used anti-VEGF agents are aflibercept, bevacizumab, and ranibizumab [6,7]. Intravitreal therapy improves the ocular prognosis of DME patients but requires regular follow-up for a long period (years), with a high burden for patients, caregivers, and the healthcare system. While DME’s clinical and pathophysiological aspects are widely studied, patients’ perception of their disease is underestimated. This study aimed to understand the real healthcare pathway for DME from the patient’s perspective and not in terms of theoretical guidelines [8]. Process mapping is the methodology used to reconstruct the path the patient takes throughout the disease and its treatments, defined as the “patient journey” [9]. One of the innovative aspects of the process mapping is that the path is not built according to a typically top-down approach (by a person with managerial skills outside the care path) but a bottom-up approach (by those who directly live the experience of illness and assistance, i.e., patients, caregivers, and healthcare professionals, who can analyze the entire sequence of assistance activities). The outcome of the patient journey mapping is represented by a flowchart that illustrates the patient’s path, providing the possibility to understand the critical areas and work on possible resolutions to improve the management of the disease [10].

## 2. Materials and Methods

### 2.1. The Patient Journey Map

The first phase of the study was the patient journey mapping, developed by an Italian committee representative of the main specialistic discipline with expertise in the treatment of DME (diabetology, ophthalmology, nursing) during a 1-day national workshop, held by the Institute Directional Study (ISTUD) Foundation. The study involved 4 centers specialized in the medical retina: the Departments of Ophthalmology at the Universities of Padova, Palermo, and Milan (S Raffaele University Hospital) and the IRCCS Bietti Foundation in Rome. The multidisciplinary board of health professionals identified the objectives of the project and the participating ophthalmology centers, validated the research methodology and investigation tools, and performed the scientific supervision of the contents and the revision of the investigation tools. 

### 2.2. Participants and Recruitment for the Narrative Medicine Phase

The second phase of the study took place in the healthcare context of the involved centers from October 2016 to November 2017. The study included patients with DME undergoing intravitreal injection therapy and their caregivers. The inclusion was voluntary to ensure the randomization of patients and caregivers. The interviews with patients and caregivers took place separately to allow free talk without conditioning. The talks lasted around twenty minutes and were intentionally focused on the relational, emotional, and affective perceptions of the participants, according to the narrative medicine (NM) approach. The narrative medicine approach was developed as a theoretical and operative approach that has been increasingly discussed in recent years. This approach aims to introduce into daily medical practice the use of narratives as a tool to collect and interpret information on the patient’s experience of illness. Narrative medicine aims to accompany the patient by listening to her/his story of illness [11]. The total number of initially planned narratives was 80 patient and 40 caregiver stories according to the principle of data saturation, which is proper protocol of qualitative research that is shared by grounded theory, which provides sample numerosity as the result of an iterative analytic process of subsequently collected data [12]. This activity did not interfere with the daily clinical practice of the treatment center, did not alter the normal visit process, and did not produce additional costs for the host facility. All research activities were carried out according to the Declaration of Helsinki after the signing of the informed consent. Ethics approval for this study was obtained from the Ethics Committee of Ospedale Università Padova (approval number: 4278/AO/17).

### 2.3. Data Collection and Narrative Analysis

The collected stories were independently analyzed by at least two blinded researchers from the ISTUD Foundation to understand the dominant and peculiar features of the text. A quali-quantitative tool, characterized by the first set of multiple-choice questions, followed by a subsequent narrative plot, was drawn up. The main items investigated through the tool were sociodemographic aspects, path in care, impact on everyday life, care, burden of illness, and perspectives for the future. For the quantitative answers obtained from the questionnaires and narratives, traditional descriptive statistical analysis methods were used, which were provided for the creation of tables, histograms, and graphs. The qualitative data were examined in aggregate form, identifying clusters and quantifying recurrences using dedicated software (Nvivo 11, QSR International, Melbourne, Australia) [13]. The narratives were also reported in non-aggregate form without any reference to proper names, places, and drugs to ensure anonymity. 

## 3. Results

### 3.1. The Patient Journey

The patient journey mapping, elaborated by the healthcare professionals, was divided into three charts: (1) Chart S1: From symptoms to access to care (2) Chart S2: Definition of the diagnosis and therapy (3) Chart S3: Follow-up and changing therapies (See Supplementary Materials—Charts S1–S3). 

### 3.2. Sociodemographic Characteristics of DME Patients and Caregivers

The study collected a total of 106 narratives, 82 from patients with DME and 24 from caregivers. The demographic data of participants interviewed are summarized in Table 1. 

### 3.3. Diagnosis of Diabetes and Its DME Complication

**Figure 1:**
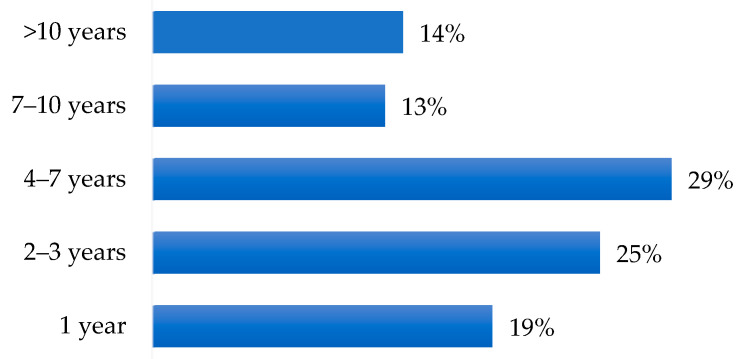
Diagnosis of DME (years after diagnosis of diabetes).

Most patients discovered diabetes through routine blood tests or the onset of symptoms. The time elapsed from the appearance of macular edema to the consequent diagnosis was 5 years on average (Figure 1). 

Both eyes were generally affected (71% of cases). In 18% of cases, only the left eye was affected and in 11% of cases, only the right eye was affected.

With regard to the degree of retinopathy: 48% of patients didn’t know or remember whether it was proliferative or non-proliferative retinopathy, while the remaining 52% were known to be affected by diabetic retinopathy (proliferative (33%) and non-proliferative retinopathy (19%).

### 3.4. The Impact of DME on Daily Life

One of the main aims of the study was to collect the impact of DME on the quality of life of the people affected by this complication of diabetes. 

**Figure 2:**
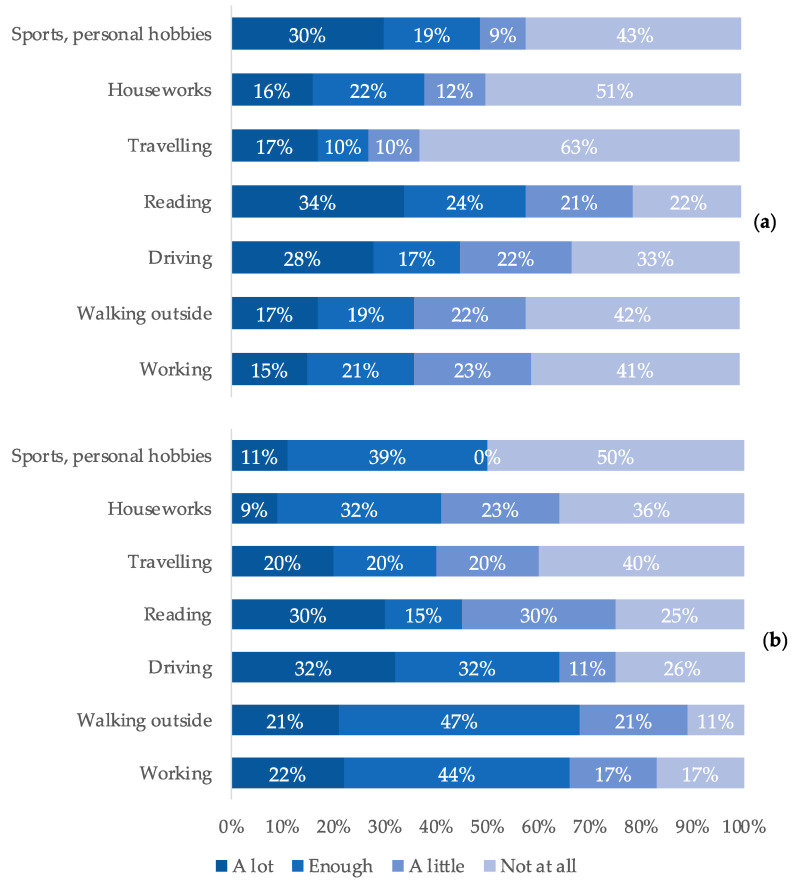
Difference in the perceptions of patients (**a**) and caregivers (**b**) regarding the limitations of affected patients in daily activities.

The activities that suffered the most from the disease were, according to patients: reading (34%), hobbies (30%), and driving (28%) (Table 2). Thirty-six percent of patients declared a negative impact on activities such as walking and working (Figure 2). 

Human relationships remained good for patients overall. Driving became difficult for 45% of the patients surveyed, while 64% of caregivers surveyed reported that, according to their perception, driving was difficult for the affected patients. However, 72% of patients never felt sad because of this complication (55% of the interviewees) or only rarely (17% of the interviewees), despite more than half of the patients feeling worried (51% of the interviewees) because of DME. A large proportion of the interviewees felt more nervous (46% of patients) due to reduced autonomy. 

### 3.5. Treatment of Diabetes and Its Eye Complication 

The most frequent combination in DME treatments was represented by the alternation of intravitreal injections and laser treatments (40 cases, 49%). On average, patients receiving anti-VEGF underwent 12.5 injections. In almost all cases (95%), patients easily remembered the number of intravitreal injections they had been subjected to but had difficulties remembering the nature of the injected substances (whether steroids or anti-VEGF). Ten percent of patients did not retrace their therapeutic planning. Over 70% of respondents did not find it difficult to accept diabetes and its complications but a percentage of patients had difficulties maintaining an adequate and stable blood sugar level (26%). 

In most cases, patients always came to medical examinations and followed the treatment (84% for both items). The indications of the ophthalmologist were respected by 58% of patients while only 15% declared that they respected the diabetologist’s indication. Half of the respondents expressed their gratitude for the assistance received (50%); in some cases, patients were required to improve the relational aspect, in particular the ability to communicate clearly with simple language without limiting the clinical aspects (22%) and to implement and improve the general organization of the departments (19%). Patients reported increasing collaboration between diabetes healthcare professionals (Table 3).

**Figure 3:**
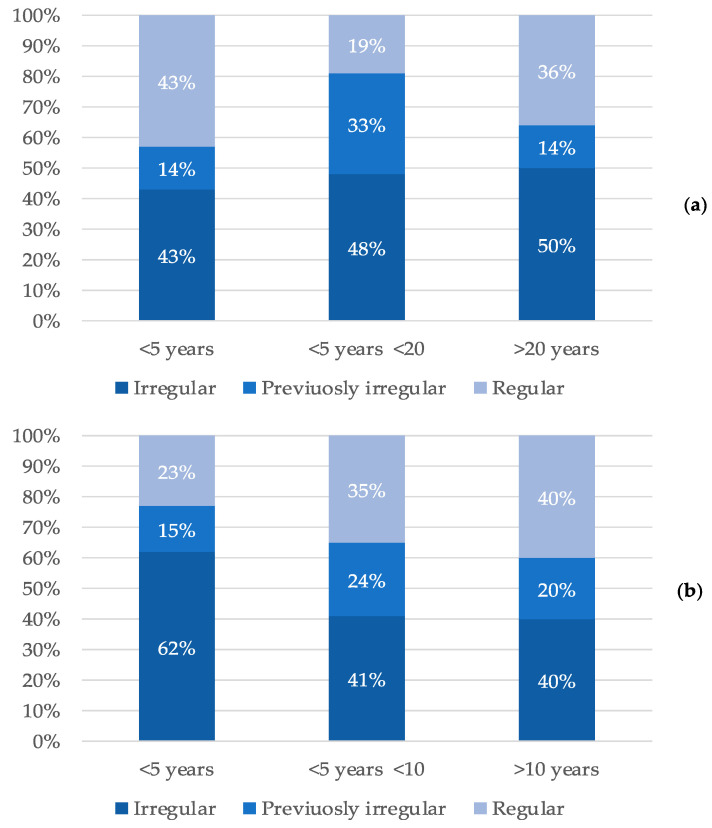
Behavior of patients in the management of diabetes as a function of the time elapsed since the diagnosis of diabetes (**a**) and DME (**b**).

The narratives showed that patients’ management of diabetes had often been neglected in the past and then they became diligent because of the worsening of the eye complication. Almost half of the respondents were irregular (49%) or had been in the past (23%) in the management of diabetes (Figure 3).

**Figure 4:**
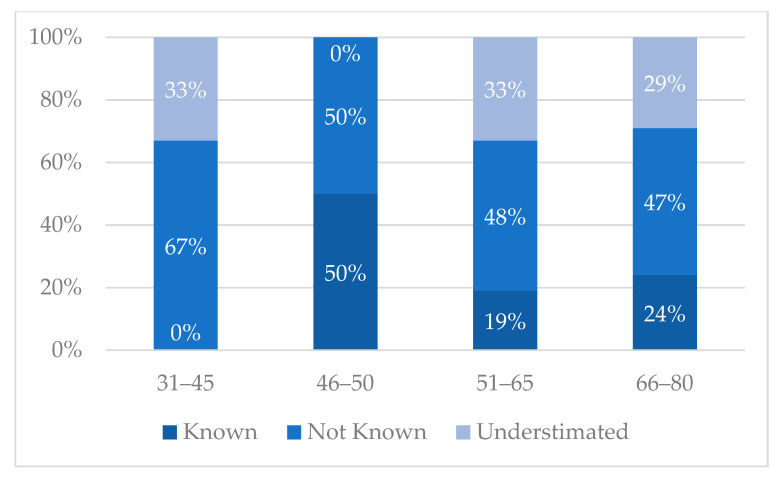
Knowledge of the complications of diabetes.

**Figure 5:**
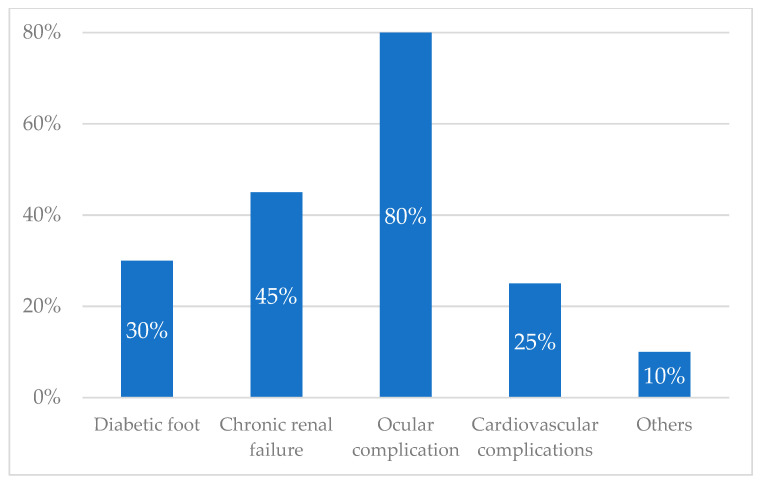
Most feared complications of diabetes.

From the narratives, it emerged that there was a lack of knowledge about the complications of diabetes. Forty-eight percent of patients didn’t know that diabetes could seriously compromise other organs leading to complications, while 31% underestimated them. Twenty-one percent were previously informed about complications. The complications that most frightened the interviewed patients were the ocular ones (almost 80%) (Figure 4 and Figure 5 and Table 4). 

As for medical examinations, patients declared having difficulties in terms of costs, the booking process and reconciling the management of the disease with other commitments. In general, patients lived far from the treatment center, 100 km away on average, and spent about six h away from home because of the visits (Table 5 and Table 6). The main activities provided by caregivers were accompanying the patient to medical examinations (79%) and supporting them in daily activities (52%) due to the reduced possibility of driving. Considering that 56% of caregivers were employed, accompanying family members to visits represented a social cost in terms of lost earnings or permits. As for the future, it is interesting to underline that only 3% of respondents were optimistic about the future. In many cases, patients were concerned or pessimistic (28%), while 24% of interviewees didn’t think about it, living in the present day by day. 

## 4. Discussion

DME is the major cause of vision loss associated with DR, the most common complication of DM, and represents one of the leading causes of visual impairment and preventable blindness worldwide in the adult working population [14].

While the clinical and pathophysiological aspects of DME are widely investigated, the patient’s perception is generally underestimated [15]. This study allowed a group of Italian clinicians with expertise in the treatment of DME to better understand the healthcare pathway for DME not in terms of theoretical guidelines but from the patient’s perspective. If the consultation provided all the stakeholders involved in the management of DME with patient journey flowcharts to refer to, the narrative medicine approach added emotional information. 

According to the narratives, patients have to reduce daily activities such as reading, personal hobbies, and driving. The loss of personal autonomy, emblematically expressed through the limitations of driving, forces patients to ask and depend on others and leads to discomfort and a risk of depression (“I’m 59 years old and if they take away my driving license, I’m in trouble”, Table 2). This confirms the correlation between visual impairment and decreased quality of life reported in the literature [16,17].

The loss of personal autonomy engages caregivers in accompanying patients to medical examinations and supporting them in errands with an associated social cost in terms of lost earnings or permits (“The biggest thing is that you can’t take two steps on your own”, Table 2). 

The Italian Health Service system estimates that 10 billion euros are spent annually caring for patients with diabetes [18]. Diabetic retinal involvement affects approximately one-third of diabetic subjects and ocular complications emerge as the most feared ones (“I did not believe that diabetes could do all this damage to vision, to the eyes, … I am especially concerned about those”, Table 4) [19]. The main risk factors associated with an early onset and rapid evolution of DR are the duration of DM, poor glycemic control, and the presence of concomitant arterial hypertension [20]. From the narratives, it emerged that awareness about diabetes and its complications is very limited (“I didn’t know the complications of diabetes. For me my only knowledge was not to eat sweets, stop”, Table 4). This results in a diagnostic delay, greater visual impairment, and a higher social cost. This finding highlights the critical role of patient−clinician communication and the need for campaigns to improve the awareness of DME strategies among target patient populations. Recently, we have assisted in a huge development of screening tools and programs for DR, more than what has been produced for any other retinal disease [21]. However, patients’ awareness of DR relevance and screening value is the first step toward successful screening programs and disease management [22].

Moreover, some of the main difficulties complained about by patients in accessing care concerns the organization of the treatment path, the times and costs of transfers to treatment centers, and the hours of waiting (“I have a lot of difficulties because the waits are long”; “…eye complication costs a lot to me, so it impacts”, Table 5). Therefore, strategies to improve the patient’s journey should include the development of local protocols for easy access of patients to the correct pathway of care (See Supporting Information—Supporting Information 1 and 2). People with diabetes and DME and their caregivers are generally grateful to professionals working in eye care centers. As qualities to be enhanced, patients indicate relational ones: they insist that all health professionals, doctors, and nurses, have the capacity to be more empathic, more “human”. From here emerges the need to advise the ophthalmology community about the necessity of correct communication with patients for better compliance and reduced discomfort. 

A patient rightly defines diabetes as “it is all a whole” to indicate the systemic vision of the disease that cannot be treated in “pieces” by each specialist, but only through an integrated multi-specialist and multidisciplinary care path (the full holistic approach). In the management of DME, there is no focus on the individual sphere (illness) and there is often a gap in the multidisciplinary approach with other health professionals such as psychologists, therapists, and rehabilitators. Therefore, narrative medicine allows us to explore the universe of illness in its psycho-emotional articulation and focuses on the inseparable continuum that links the condition to the person: in this sense, it captures the evolution towards a holistic conception in which the pathology is no longer separated, but an integral part of the patient. A holistic approach could improve the healthcare pathway both in terms of disease management (ophthalmologic problems) and the emotional sphere. The NM method of analysis provides qualitative and emotional patient-guided information not addressed by most quantitative surveys on DME to improve awareness of each step of the healthcare pathway in DME [23,24].

## 5. Conclusions

DME may reduce the quality of life of affected patients, both from a functional and psychological point of view. Although the clinical aspects of DME have been extensively studied, the patient’s perceptions are often disregarded. The narrative medicine approach highlights some critical points in the healthcare journey of DME patients. It provides qualitative and emotional patient-guided information generally not addressed by the surveys on DME, representing a useful background for the implementation of patient management algorithms and pathways of care [25]. 

## Figures and Tables

**Table 1:** Participants’ demographic and clinical data.

Number of Participants	DME Patients N = 82	Caregivers N = 24
Male	57%	39%
Female	43%	61%
**Age (years)**		
Average	65	55
Range	30–82	22–80
**Type of diabetes**		
DM I	17%	
DM II	83%	
**Duration of diabetes (years)**		
Mean	19	
Range	0–58	
<10 years 10–20 years >20 years	24% 40% 36%	
**Age at diagnosis of diabetes (years)**		
DM I		
Average Range	31 11–50	
DM II		
Average Range	48 22–73	
**Marital status**		
Married	88%	75%
Unmarried	12%	25%
**Instruction level**		
Obligatory education	65%	57%
Diploma/degree/master	35%	43%
**Occupational status**		
Employed	40%	56%
Retired/unoccupied	60%	44%
**Population of interviewees’ hometown (amount of people)**		
<25,000	40%	
25,000–250,000	20%	
>250,000	40%	
**Relationship**		
Partner		42%
Son/daughter		39%
Others		19%
DM: diabetes mellitus		

**Table 2:** Impact on quality of life.

Driving	“In everyday life I have problems driving at night: when I drive, I look fixed on the strips, otherwise I have the flash in my eyes“ “I think about the driving license, it expires on the 23rd and I am very worried, that is a thought unfortunately” “I’m just sorry that I had to abandon the car both for the problem that I do not see there and for the foot that I no longer have the reflex ready” “The only problem I have right now is that I have everything expiring. The driving license, the carrying of weapons and I must redo everything and with the problems that there are in Italy it becomes difficult. I’m 59 years old and if they take away my driving license, I’m in trouble” “Yes, I am sad because I am afraid of losing my autonomy, I do not want to depend on anyone. If I had to know that tomorrow, they take away my car (crying) because it’s like dying”
Reading	“Reading I struggle because closely I can’t, it’s a still image, I can’t adjust, focus” “Before I read a lot, the grandchildren gave me books to read the stories but now I can no longer read”
Walking	“I no longer feel safe walking, I stumble often, the doctor said it is a beginning of diabetic neuropathy” “I don’t have the confidence to walk quietly alone, balance, being able to visualize people’s faces. The biggest thing is that you can’t take two steps on your own”
Hobbies	“First I sewed with crochet, now not” “I used to play bowls and I don’t see the ball far away anymore” “The eye problem affects my day, I loved sewing, knitting, reading…now I struggle if there is a long word I struggle and so I go to the meaning of the sentence but maybe I understood in one way and instead there was written another thing, and everything changes. I can’t stick the needle even with the needle thread and then I get angry and cry there”

**Table 3:** Lack of the multidisciplinary approach.

“Diabetologists and ophthalmologists do not know each other, I am the one who acts as an intermediary. It would be necessary that on the same day that you visit here, you could do an hour even with the diabetologist, it would be very important“ “Diabetologist and ophthalmologist do not dialogue, I had to report to the diabetologist, and he verifies everything. They are two worlds that do not talk to each other“ “Having a fixed point of reference would be appropriate. We are the means to make cardiology, diabetology, and ophthalmology talk to each other. We always bring the reports, for example, we brought to the diabetologist what we did with the cardiologist. Everyone looks at his specialty, there is no real coordination point, he updates the computer” “However, diabetologist and ophthalmologist do not talk to each other, nor does one ask about the other… they are a number for both. Perhaps the doctors have lost sight of the patient but not through his fault but because he is overloaded” “The problem is all a whole. Diabetes itself is not disabling but it is the problem that creates you”

**Table 4:** Awareness of diabetes complications.

Didn’t know	“I did not know the complications of diabetes, I realized it 5 years ago that I began to see badly, so I had a visit” “I did not know about the visual complication, they told me that diabetes brings complications on other organs, but I found out only after that I could get to this” “I didn’t know the complications of diabetes. For me, my only knowledge was not to eat sweets, stop” “When I was diagnosed with diabetes I did not know about the complications, I found out a few weeks later. Surely the one that scares me the most is not seeing us anymore“ “When I was diagnosed with diabetes, I didn’t know the complications, I discovered them as we went on, eye complications are the ones that scare me the most”
Underestimated	“I knew the complications of diabetes, but I honestly ignored the visual ones, I didn’t think they were so invasive” “I did not believe that diabetes could do all this damage to vision, to the eyes, … I am especially concerned about those”

**Table 5:** Distance to reach the clinic and time spent to treat diabetic retinopathy.

Average km to reach the Diabetic Eye Clinic	102 km
Average time taken to reach the Diabetic Eye Clinic	50 min
Average monthly cost of reaching the Diabetic Eye Clinic	34 euros
Average time taken to perform the ophthalmological evaluation at the hospital (including the travel)	5.8 h

**Table 6:** Main issues in treatments.

Economic burden	“This disease impacts economically: I take 860 euros from the government: total 860 euros and eye complication costs a lot to me, so it impacts” “Economically the management of diabetes and vision has affected economically, I manage everything privately. Fixing a visit and an OCT is impossible and therefore you are forced to this”
Distance to the diabetic eye clinic	“Not having nearby clinics, I must move here in the mayor diabetic clinic., the trips impact” “To come here I come by car and my son takes me” “To come here, most of the time I come by public transport: I have the bus under the house, then I take the train” “If I do not have to do the eye examination I come by car, it takes 30 min (20 min if I come by car)” “It’s 50 km each time to come here”
Time spent for the visits	“When I leave the house to when we leave here passes a long time, es Friday that they did not do the injections I arrived at 8 and I left here at noon and a half” “Today it was a bit longer, but I usually stay here two or two and a half hours” “It takes half a day for an examination that lasts 3 min. It is from one that I am here, now that I am out of here it will be 4 and a half. Now that you go to the acceptance that even if you arrive well in advance, however, you lose 3⁄4 of an hour, then go upstairs, you must wait for them to call you, make another line an hour and a half …“
Difficulties in reservations	“For reservations I have a lot of difficulty because the waits are long”

## Data Availability

The data presented in this study are available in the article.

## Supplementary Materials

The following supporting information can be downloaded at: https://www.mdpi.com/article/10.3390/ijerph19159367/s1.
