# Supplementary figures and images for: The Narrative Medicine Approach in the Treatment of Diabetic Macular Edema: An Italian Experience
Source: Int J Environ Res Public Health. 2022 Jul 30;19(15):9367. doi: 10.3390/ijerph19159367 (PMC9368569; doi:10.3390/ijerph19159367)

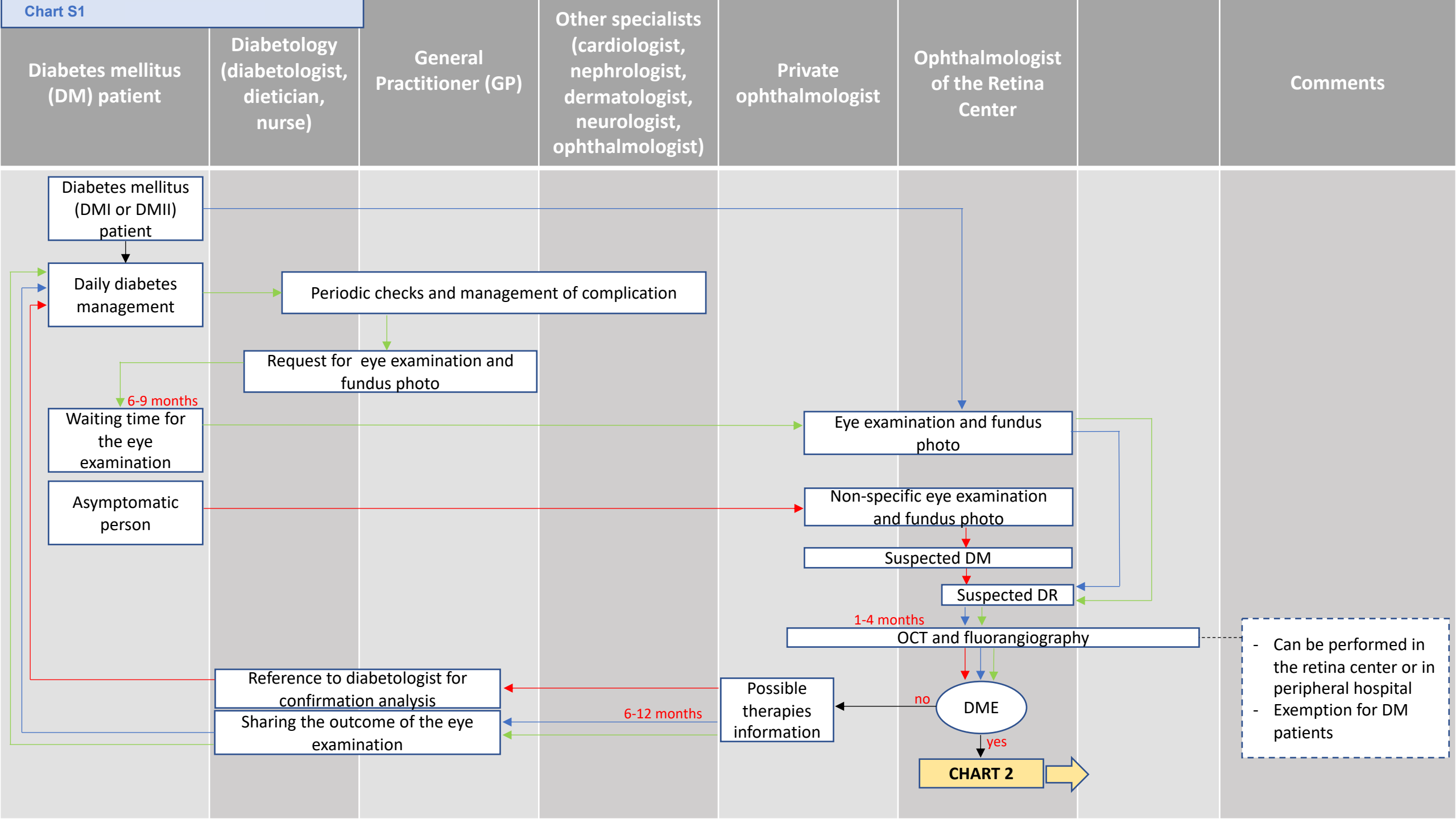

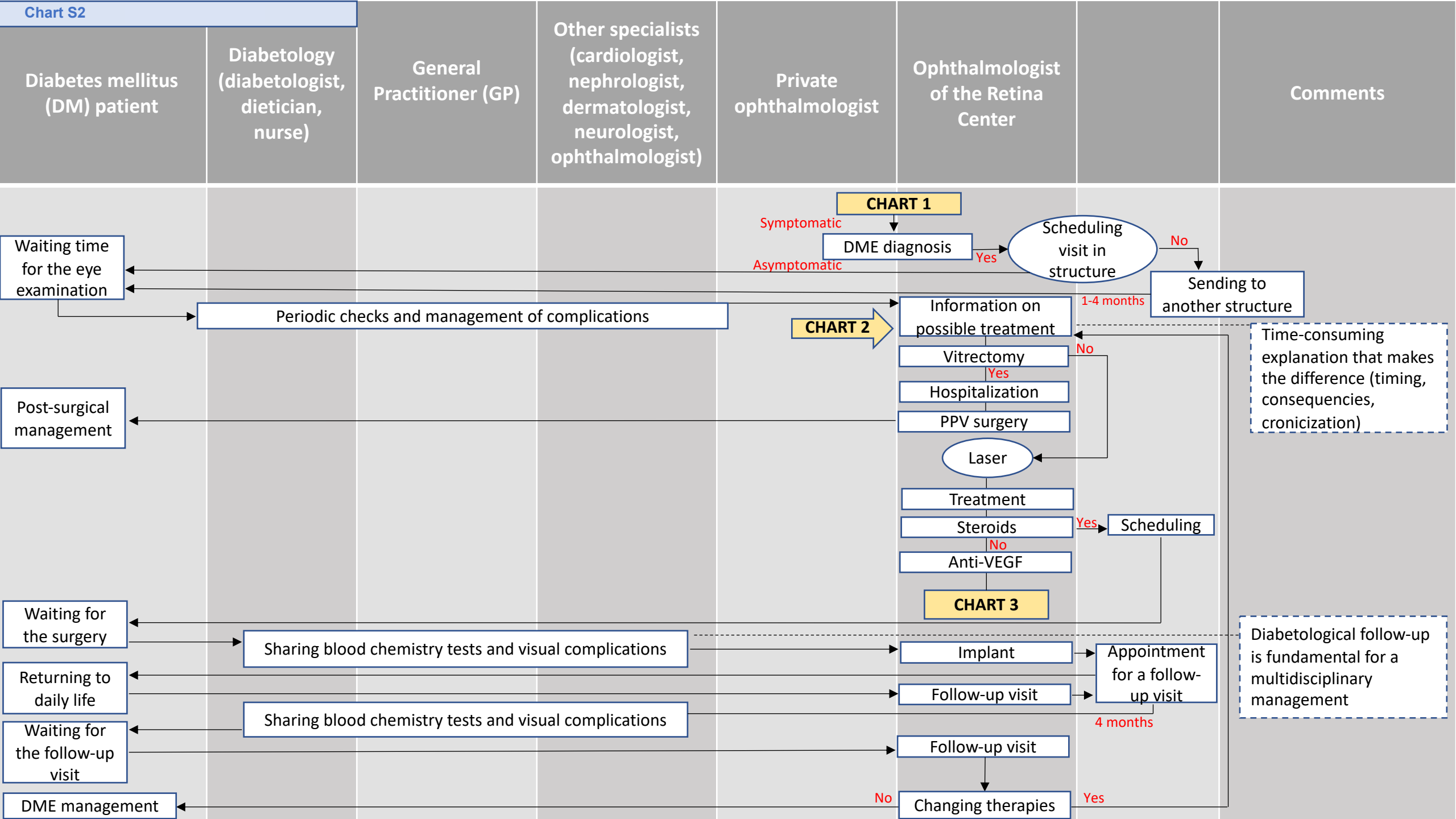

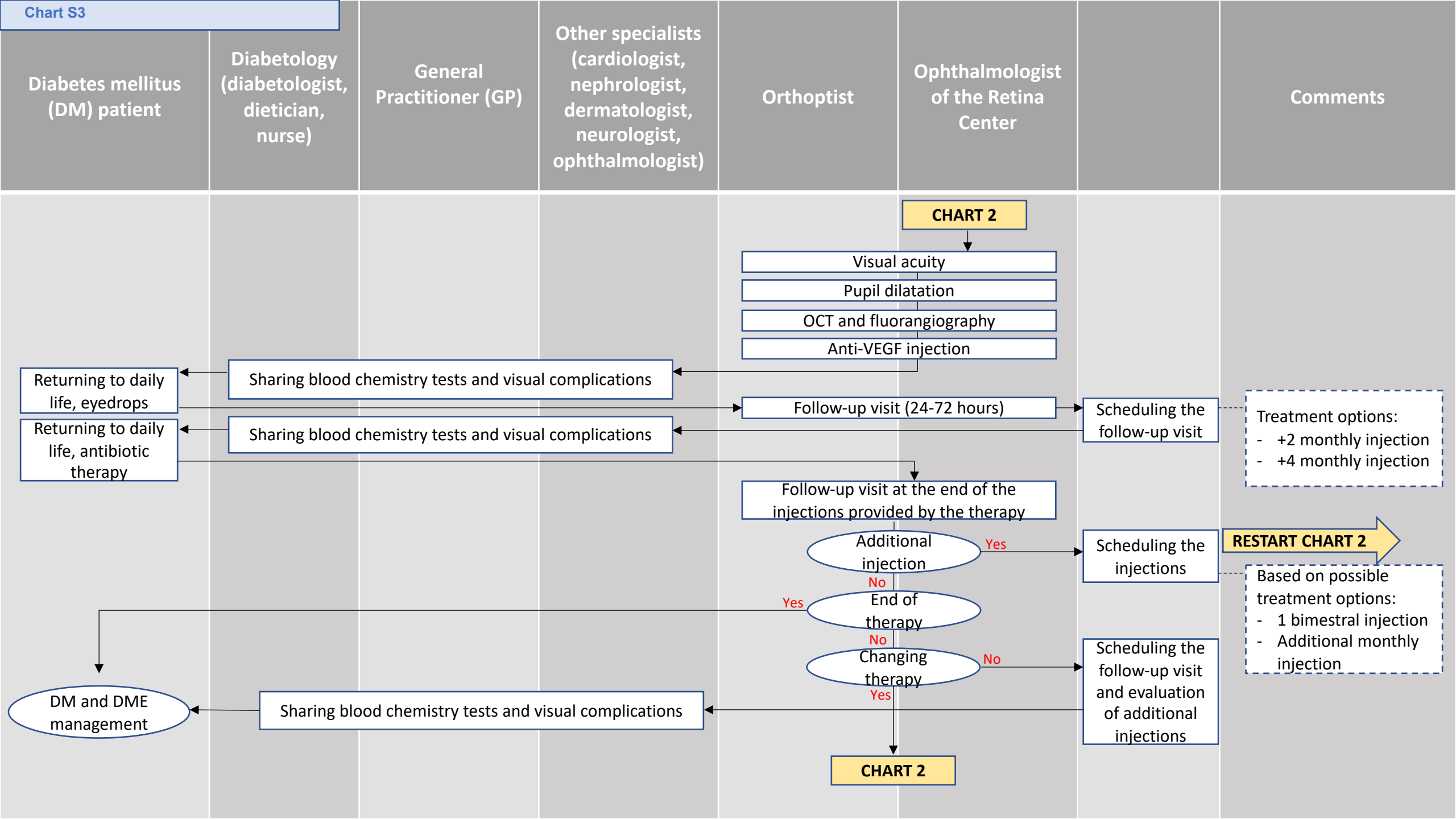

Supplement: Supplementary file 1 [file ijerph-19-09367-s001.zip › ijerph-1802928-supplementary.pdf]
